# Positive effect of septimeb™ on mortality rate in severe sepsis: a novel non antibiotic strategy

**DOI:** 10.1186/2008-2231-20-40

**Published:** 2012-09-25

**Authors:** Kaveh Eslami, Ata Mahmoodpoor, Arezoo Ahmadi, Mohammad Abdollahi, Koorosh Kamali, Sarah Mousavi, Atabak Najafi, Maryam Baeeri, Hadi Hamishehkar, Leila Kouti, Mohammad Reza Javadi, Mojtaba Mojtahedzadeh

**Affiliations:** 1 Faculty of Pharmacy and Pharmaceutical Sciences Research Centre, Tehran University of Medical Sciences, Tehran, Iran; 2 Faculty of Pharmacy, Ahvaz Jondishapour University of Medical Sciences, Ahvaz, Iran; 3 Department of Anesthesiology and Critical Care Medicine, Faculty of Medicine, Tabriz University of Medical Sciences, Tabriz, Iran; 4 Department of Anesthesiology and Critical Care Medicine, Sina Hospital, Faculty of Medicine, Tehran University of Medical Sciences, Tehran, Iran; 5 Department of Public Health, School of Public Health, Zanjan University of Medical Sciences, Zanjan, Iran; 6 Clinical Pharmacy Department, Faculty of Pharmacy and Research Center for Rational Use of Drugs, Tehran University of Medical Sciences, Tehran, Iran; 7 Clinical Pharmacy Department, Faculty of Pharmacy, Tabriz University of Medical Sciences, Tabriz, Iran

**Keywords:** Severe sepsis, Immunomodulation, Septimeb, ICU

## Abstract

**Background:**

Septimeb is a new herbal-derived remedy, recently approved for its potential immunomodulatory effects. Regarding the key role of immune system in the pathogenesis of severe sepsis and lack of any standard treatment for improving survival of these patients; we evaluated the effect of Septimeb -as an adjutant to standard treatment-on inflammatory biomarkers and mortality rates in patients with severe sepsis.

**Methods:**

In this multicenter, randomized, single-blind trial, we assigned patients with severe sepsis and Acute Physiology and Chronic Health Evaluation (APACHE II) score of more than 20 to receive standard treatment of severe sepsis (control group) or standard treatment plus Septimeb. This group was treated with Septimeb for 14 days then followed up for another14 days. APACHE score, Sequential Organ Failure Assessment (SOFA) and Simplified Acute Physiology Score (SAPS) were calculated daily. Blood samples were analyzed for interleukin 2 tumor necrosis factor-α, total antioxidant power, platelet growth factor and matrix metalloproteinase 2.

**Results:**

A total of 29 patients underwent randomization (13 in control group and 16 in Septimeb group). There was significant difference between the Septimeb and control group in the 14 days mortality rate (18.8% vs. 53.85 respectively, P=0.048). Compared to control group, Septimeb was significantly effective in improving SAPS (P= 0.029), SOFA (P=0.003) and APACHE II (P=0.008) scores. Inflammatory biomarkers didn’t change significantly between the two groups (P>0.05).

**Conclusion:**

Septimeb reduces mortality rates among patients with severe sepsis and it could be added as a safe adjutant to standard treatment of sepsis.

## Introduction

Sepsis is one of the most prevalent and fatal diseases in Intensive Care Units (ICU) resulted in 30-60% mortality [1,2]. Sepsis is a systemic inflammatory response to an infection leading to endothelial dysfunction, impairment of microcirculation, tissue hypoxia, apoptosis and finally multiple organ failure and death [3]. Nowadays the role of inflammatory cytokines, oxidative stress and immune system in the pathogenesis of sepsis is obvious. Continuous elevated levels of various cytokines in severe sepsis could result in uncontrolled inflammation [4-7]. Breaking of inflammatory cascade may lead to improving of survival [8]. A significant association exists between inflammatory cytokine levels in the first 72 hours of severe sepsis and severity of tissue hypoxia, organ dysfunction and mortality [9]. IL-6 and Acute Physiology and Chronic Health Evaluation (APACHE) II score are used respectively in prediction of mortality and clinical evaluation of severe septic patients [10]. Therefore modulation of inflammation and immune system response could be one of the strategic plans to manage sepsis.

Several immunomodulatory therapies have been evaluated in sepsis. Corticosteroids [11,12], anti Tumor Necrosis Factor α (TNF α) antibodies [13,14], Anti Interleukin 1 antibodies [15], Platelet Aggregating Factor (PAF) antagonist [16,17], antioxidants especially selenium [18,19], modulation of nutrition [20], modulation of coagulation [21] and complement pathway [22] are examples of immunotherapy in sepsis, but none of them were effective on survival of patients. Procedures such as plasmapheresis and hemofiltration slightly improve prognosis of sepsis by reducing circulating levels of inflammatory cytokines but couldn’t decrease mortality rate [23].

Analysis of current immunomodulating strategies indicates that monovalent approaches in isolation are unlikely to restore immunostasis or attain status of complete therapy. It is likely that multiple immunomodulating strategies will be necessary to achieve clinical success owing to complex interplay between pathways. Botanical extracts provide cytoprotective, antiinflammatory, and antimicrobial activities in addition to immunoregulatory activity [24].

Septimeb (a more potent form of IMOD^TM^) is a herbal extracts including Tanacetum vulgare (tansy), Rosa canina and Urtica dioica (nettle) in addition to selenium, flavonoids and carotenes. Tanacetum vulgare may relieve anti-inflammatory symptoms and Rosa canina defers blood glucose and cholesterol elevation. Extracts from Urtica dioica may prevent maturation of myeloid dendritic cells and reduce T cell responses [25]. Septimeb can regulate TNF-α, interferon-γ (IFN-γ) and IL-2 [26]. IMOD has been patented in Europe with code of WO/2007/087825 for its immunomodulator and anti-TNF-α capacities and improving CD4 in HIV positive patients [27,28]. The pre-clinical safety studies of IMOD in animals and phases I and II trials have been successfully conducted, showing optimistic results [29,30]. As sepsis is an immune mediated disease and modulating of immune system might be effective in improving prognosis and survival, we aim to evaluate Septimeb’s effect on plasma levels of inflammatory biomarkers, and mortality rates of patients with severe sepsis.

## Methods

### Patient selection

In all cases, informed consents were received from patients or their legal guardian. The study procedure and protocol were approved by the ethical committee of Tehran University of Medical Sciences (TUMS). Our clinical trial was registered in Australian Newzeland Clinical Trial Organization with code number of (ACTRN012607000376448).

### Study subjects

Between March 2009 and April 2011, 29 patients with severe sepsis and Acute Physiology and Chronic Health Evaluation (APACHE) scores of more than 20, admitted to general ICU of “Sina” Hospital of TUMS and “Shohada” Hospital of Tabriz University of Medical Sciences were enrolled in this study. Exclusion criteria for all patients were ages below 18 and more than 65 years, pregnancy, breast feeding, over 24 hours of sepsis diagnosis and death probability within 28 days due to nonspecific reasons (such as renal or liver failure).

Patients were randomized via block randomization to receive one of the following treatments: standard treatment for severe sepsis (control group) and standard treatment plus Septimeb (intervention group) for 14 days and were also followed up for another 14 days. According to latest international guidelines [31], standard treatments for severe sepsis were implemented as following: early resuscitation within the first 6 hours of admission, appropriate diagnosis studies to ascertain causative organisms before starting antibiotics, early administration of broad spectrum antibiotic therapy, hypotension control with avasopressor, fluid administration to target Central Venous Pressure (CVP) of 8–12 mmHg and central venous oxygen saturation (Sa O_2_) > 95%, low dose steroid, glycemic control(blood glucose <150 mg/dl), target hemoglobin values of 7–9 g/dl in absence of coronary artery disease or acute hemorrhage, lung protection ventilation and standard prophylactic measures for deep vein thrombosis (DVT) and Stress-related Mucosal Damage (SRMD).

Patients in intervention group received 240mg (4 ml) of Septimeb in 150 ml of DW 5%, infused over 1.5 hours on the first day. All hemodynamic data were monitored during the infusion and in case of any negative hemodynamic deterioration, dermal rash, urticaria or anaphylactic reaction, the infusion discontinued. Then 360mg (6 ml) of the drug was infused in 150 ml of DW5%, everyday for 14 days. The time gap between the beginnings of treatment with intervention and the diagnosis of sepsis was also noted to evaluate possible impact of the onset of intervention on mortality.

Demographic data and clinical information were obtained at the beginning (Table 1). APACHE scores, sequential organ failure assessment (SOFA), and simplified acute physiology score (SAPS) were calculated daily. Higher scores indicate more severe illness and a higher number of therapeutic interventions.

**Table 1:** Demographic and baseline characteristic of patients

	**Control group (N:14)**	**Septimeb group (N:16)**	**P value**
Age (year)	45.69 ± 20.67	39.56 ± 16.46	0.38
Male, No.	10	15	0.18
APACHE II	24.76 ± 4.14	27.56 ± 7.39	0.23
SOFA	8.92 ± 1.97	9.25 ± 2.32	0.69
SAPS	47.69 ± 11.61	43.68 ± 11.21	0.35

Data are mean ± SD. APACHE: Acute Physiologic And Chronic Health Evaluation. SAPS: Simplified Acute Physiologic Score. SOFA: Sequential Organ Failure Assessment.

Mortality during ICU stay and mortality during 14 days were recorded. The standard treatment of sepsis was continued throughout ICU stay of patients.

### Sample collection and handling

All patients had central venous catheters and arterial lines for blood sampling. For all patients there were seven scheduled time points to assess IL-2, TNF-α, total antioxidant power (TAP), Matrix Metalloproteinase2 (MMP2) and Platelet Growth Factor (PGF). The levels were analyzed via commercially available enzyme-linked immunosorbent assay kit (BenderMed system, Vienna, Austria). The first sample was taken on ICU admission and prior to initiation of the therapy. Other samples were obtained on days of 1, 2, 3, 7, 10, and 14 post therapies. Blood samples were collected into vacutainer tubes containing EDTA.

The samples were spun at 3000 × g for 15 minutes in order to remove cells and cellular debris. The cell free supernatant and plasma were stored at −80°C until the time of analysis.

### Statistical analysis

All data were expressed as mean ± SD. Univariate comparisons of baseline characteristics were performed by the independent student’s t-test for parametric data and Mann-Whitney’s t-test for non-parametric data. To assess differences between the time points in each treatment group, repeated-measure analysis of variance was done to analyze changes in levels of the biomarkers. Pearson’s chi square and Kaplan-meier (Log rank) tests analyzed the differences of survival rates among the two groups. *P*-values less than 0.05 were considered statistically significant.

## Result

Thirty patients with severe sepsis and APACHE scores of over 20 were enrolled in this study (13 patients in control group and 16 in intervention group). Demographic and baseline values for APACHE, SOFA and SAPS scores have been summarized in Table 1. The differences in age and sex were not significant between two groups (P= 0.38 and 0.18, respectively). Differences in baseline values for APACHE, SOFA and SAPS scores were not statistically significant either (Table 1).

**Figure 1:**
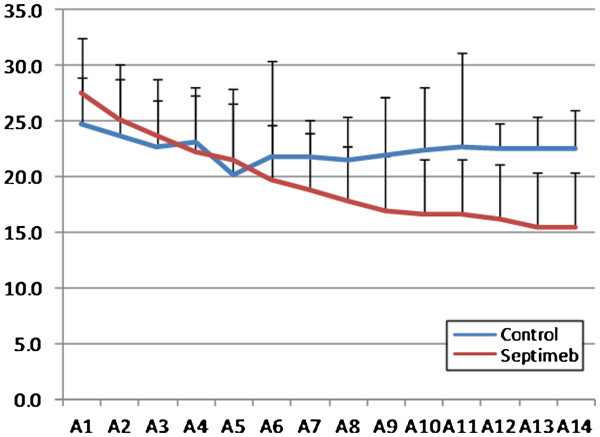
**Changes of patients APACHE II score in different days of study. **Difference between two groups is not significant (P= 0.266). A: APACHE (Acute Physiologic And Chronic Health Evaluation) 1–14: day 1–14.

**Figure 2:**
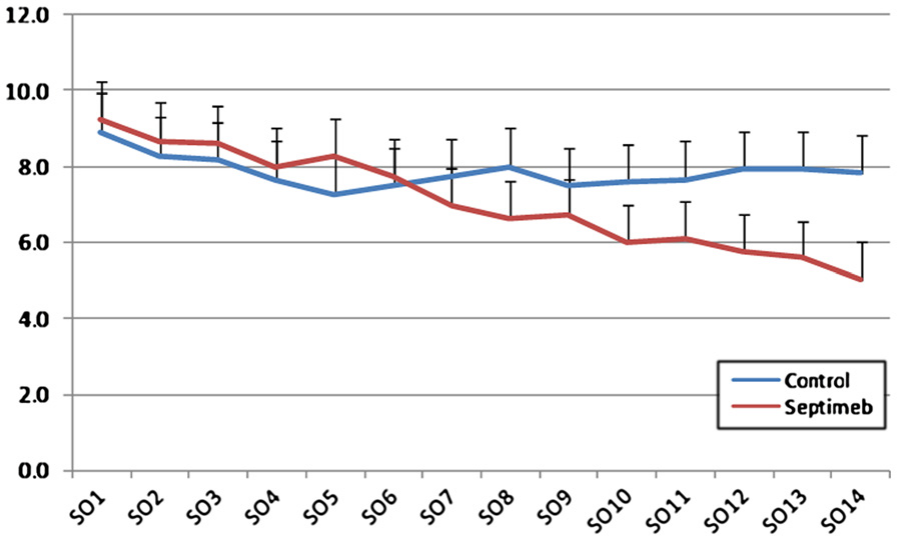
**Changes of patients SOFA score in different days of study. **Difference between two groups is not significant (P= 0.335). SO: SOFA (Sequential Organ Failure Assessment) 1–14: day 1–14.

**Figure 3:**
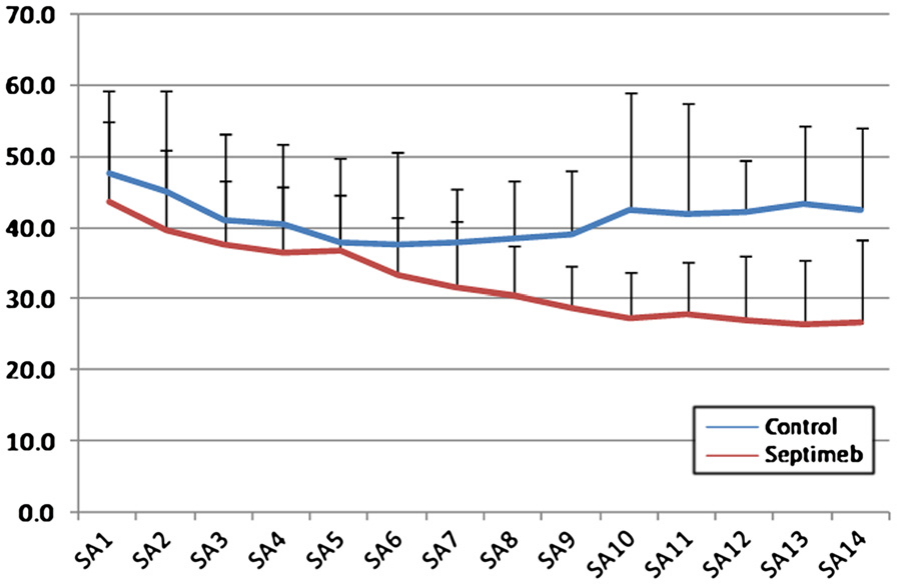
**Changes of patients SAPS score in different days of study. **Difference between two groups is not significant (P= 0.059). SA: SAPS (Simplified Acute Physiologic Score) 1–14: day 1–14.

Compared to controls, Septimeb was effective in improving APACHE (P= 0.229), SOFA (P=0.335) and SAPS (P= 0.059) scores in 14 days but the trend was not statistically significant (Figures 1, 2 and 3). But the score differences between the 14th and first day by LOCF method (Last Observation Carried Forward), showed that adding Septimeb was significantly more effective than standard treatment (Table 2).

**Table 2:** Change in patient's APACHE, SAPS and SOFA score from 1st day to 14th day study

	**Control group (N:14)**	**Septimeb group (N:16)**	**P value**
APACHE mean difference between 14^th^ and first day±SD	−1.7(±9.8)	−12.7(±7.9)	0.003
SOFA mean difference between 14^th^ and first day	−0.5(±2.3)	−4(±3.3)	0.013
SAPS mean difference between 14^th^ and first day	−3(±12.9)	−18(±16.6)	0.003

**Figure 4:**
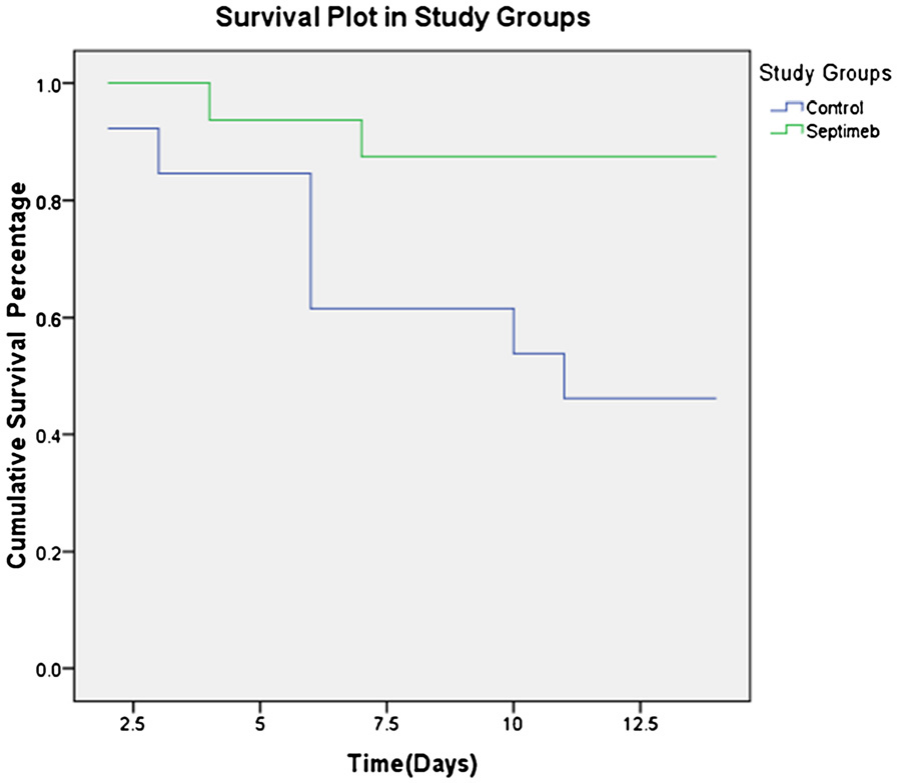
**Changes of patient’s survival during study period. **The differences in survival time was significant (P=0.019).

The mean survival time was 9.84 (7.32-12.36, CI: 95%) days in control group and 12.93 (11.49-14.3, CI: 95%) days in Septimeb group. The difference in survival time was significant (P=0.019) (Figure 4). In the intervention group, the mortality rate was 18.8% during 28 days while it was 53.8% in control group, the difference was statistically significant (P=0.048).

**Figure 5:**
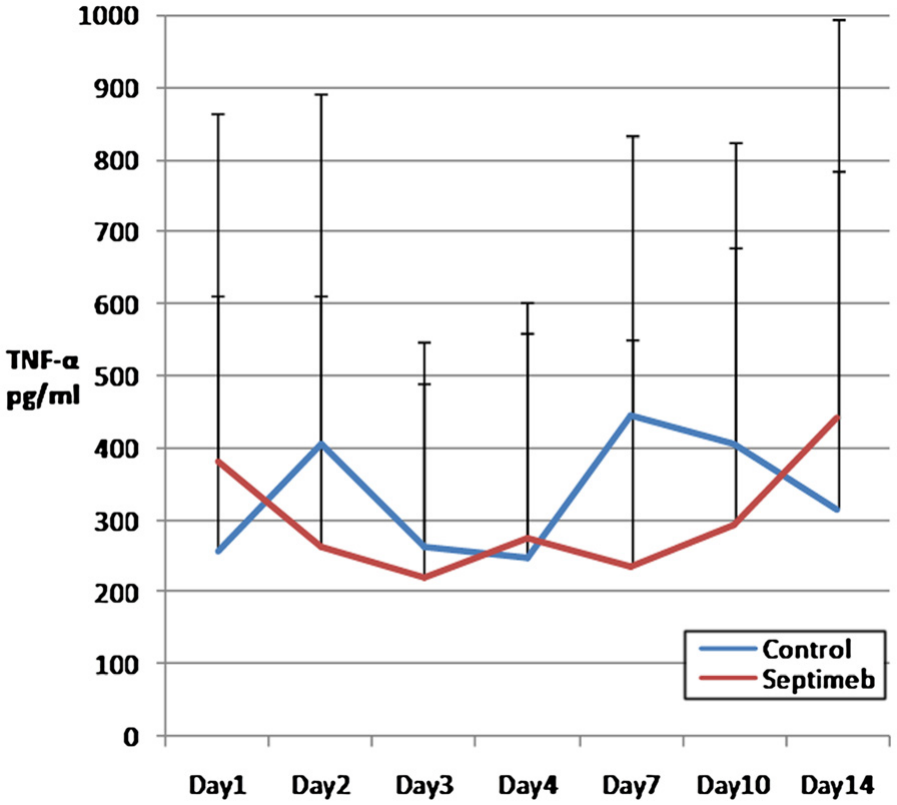
**Changes in blood TNF-α level in different days of study. **Data are mean ± SE. **Difference between two groups is not significant at P>0.05.

**Figure 6:**
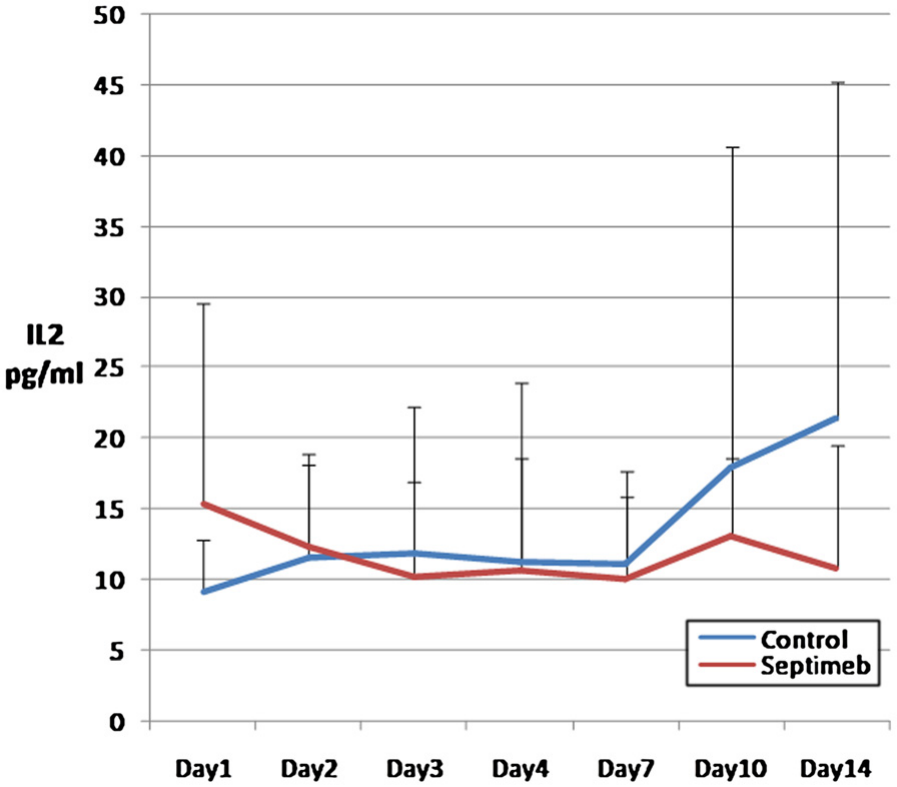
**Changes in blood IL-2 level in different days of study. **Data are mean ± SE. **Difference between two groups is not significant at P>0.05.

**Figure 7:**
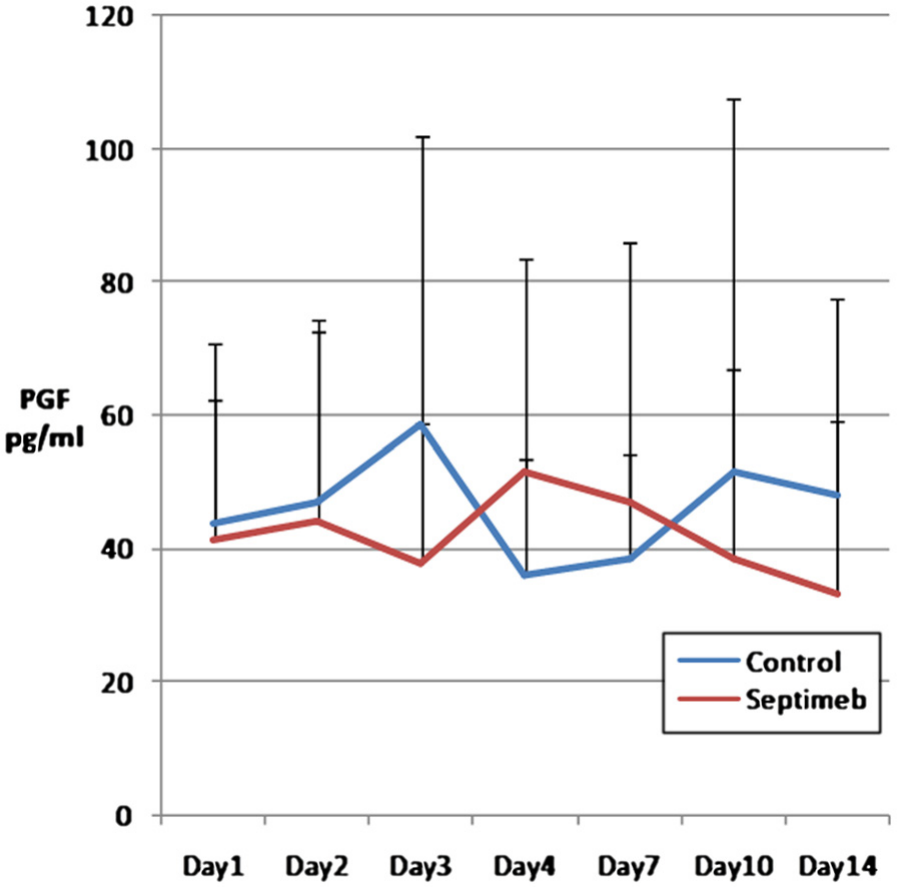
**Changes in blood PGF levels in different days of study. **Data are mean ± SE. **Difference between two groups is not significant at P>0.05.

**Figure 8:**
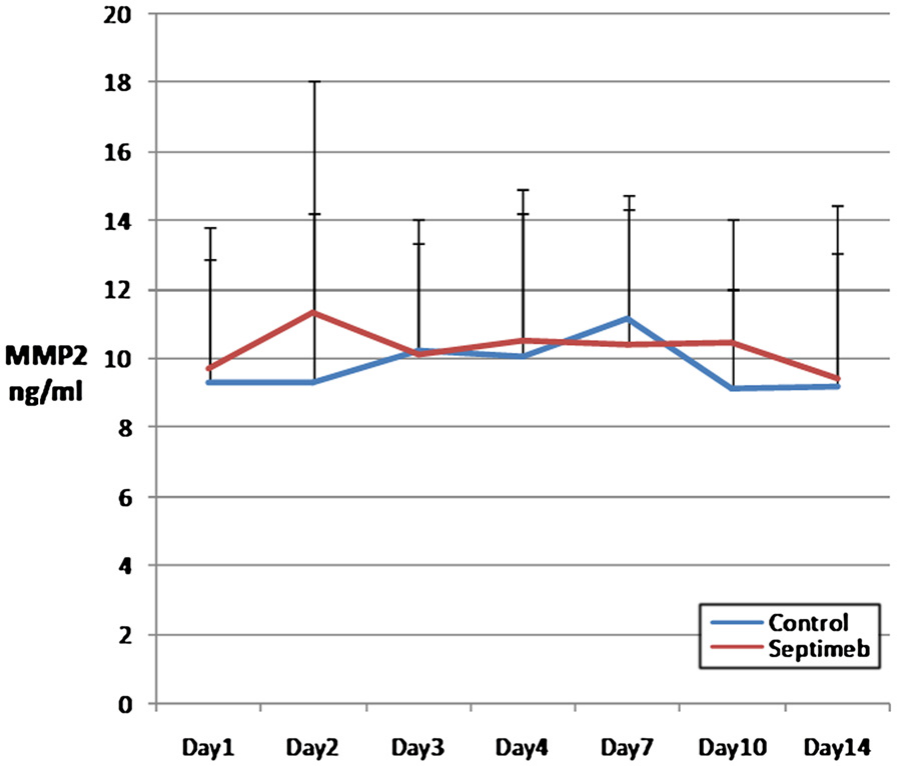
**Changes in blood MMP-2 level in different days of study. **Data are mean ± SE. **Difference between two groups is not significant at P>0.05.

**Figure 9:**
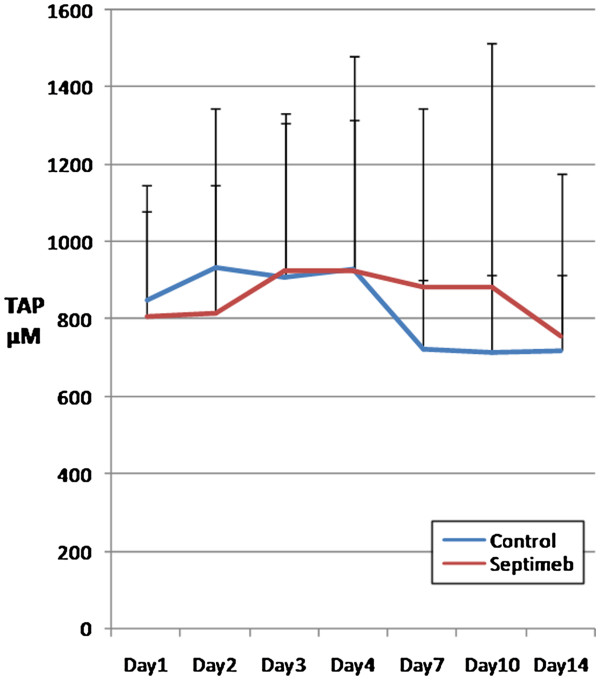
**Changes in blood TAP level in different days of study. **Data are mean ± SE. **Difference between two groups is not significant at P>0.05.

The changes in the levels of biomarkers including IL-2 (P= 0.35), TNF- α (P= 0.66), TAP (P= 0.92), MMP 2 (P= 0.62) and PGF (P= 0.45) were not different among two groups (Figures 5, 6, 7, 8 and 9). None of the time points (0,1, 2, 3, 7,10 and 14 days) showed significant differences between two groups (p>0.05).

## Discussion

The results of this study show that Septimeb, as an adjutant to standard treatment of severe sepsis could reduce mortality rates in patients, but has not significant anti-inflammatory effects.

Despite great advancements in understanding the pathophysiology of sepsis and development of novel therapeutic approaches, mortality of sepsis remains unacceptably high [32]. As mentioned earlier, immune system and inflammatory cytokines have a key role in pathogenesis of sepsis and several studies evaluated the effect of different immunomodulatory treatment on severe sepsis, but fail to improve survival of patients [11-19,21-23,33]. Among immunomodulatory agents, the activated protein C and corticosteroids are the only approved treatment for sepsis. According to the results of PROWESS trial [34,35] and a Meta-analysis [36] about the ineffectiveness of activated protein C on survival of patients with sepsis, Food and Drug Administration (FDA) withdrew this drug from the market and therefore currently there is no specific treatment for severe sepsis.

Previous studies have shown that monovalent therapies could not restore immunostasis in severe sepsis, so the need of drugs with polyvalent activities such as botanical immune-drugs seems inevitable [24,37]. Botanical immunodrugs e.g. Chinese remedy and green tea have been found to have dose-dependent attenuate bacterial endotoxin-induced HMGBI release [38,39]. Septimeb is a new herbal-derived remedy that has been suggested to be able to stimulate lymphocytes in order to produce cytokines in HIV positive patients and also inhibit TNF alpha and increase interferon gamma and Interleukin 2 [40,41]. The phase I and II trial studies with IMOD^TM^ shows this drug is safe and has no mutagenic or allergenic potentials. Based on the results of this study, Septimeb (a more potent form of IMOD) reduced APACHE, SOFA and SAPS scores, as quantitative indices of clinical status [29,30]. Improvements of these scores by Septimeb confirm the potential of this drug in alleviation of the severity of sepsis and consequently reduced mortality rates of patients.

The results of previous study [30] did not show any improvement in survival of septic patients, but in the current study Septimeb decreased mortality rate significantly, this could be because of larger sample size in this study.

Immunotherapy must be applied early during the development of immune-dysfunction following trauma or acute onset of infection, to ensure that all cellular components of immune system will be protected. There is a much earlier temporal relationship between inflammation and the onset of organ dysfunction. Mean biomarker levels during 72 hours were significantly greater in hospital non-survivors [42]. Supporting evidence showed that the initial inflammatory response directly correlates to early, but not late sepsis mortality [8]. A complex interaction of cytokines and cytokine-neutralizing molecules probably determines the clinical presentation and course of sepsis. Intervening in this sequence of events to modify the host inflammatory responses may be a beneficial treatment strategy for sepsis, but currently tested anticytokine therapies have been largely unsuccessful [20]. Levels of inflammatory biomarkers didn’t change significantly by Septimeb and positive effect of Septimeb on mortality rate might be due to other mechanisms. The main problems in severe sepsis are O_2_ extraction and O_2_ consumption. The critical importance of tissue oxygenation was addressed by a study in which patients in the earliest stages of sepsis were treated by aggressive management with fluids, blood transfusion and inotropic agents to optimize hemodynamic function [43]. After macrocirculatory resuscitation, many patients still demonstrate multisystem organ dysfunction and shock. The recent landscape of sepsis research is focusing on two separate mechanisms that may explain the persistence of the shock state. The first is the role of the microcirculation in the body, and the mixed response of various circulatory beds to the sepsis. The second is that the cells and mitochondria receiving adequate perfusion and oxygenation may still have a decreased ability to properly utilize energy to form ATP, referred to as cytopathic hypoxia [44], that can lead to profound metabolic alterations and the inability to supply energy to the tissues. These two represent attractive targets for novel adjunctive resuscitative agents in the treatment of septic shock. Therefore improving tissue oxygenation could be a possible mechanism of Septimeb in reduction of sepsis mortality.

Fortunately, Septimeb showed no adverse effects on coagulation factors like platelet count, prothrombin time, partial thromboplastin time, fibrinogen, and D-dimer. Hemodynamic instability and anaphylactic reactions were not observed during infusion. It could be a safe adjutant to other standard treatments of sepsis [45].

## Conclusion

Despite several limitations of this study, especially the small sample size, Septimeb shows positive effects on survival of patients with severe sepsis, considering the withdrawal of activated protein C from markets. Additional studies with adequate sample size is recommended to be performed with Septimeb, but regarding the results and also limited side effects of Septimeb, it could be considered as an adjutant along with other standard measures for treatment of severe sepsis.

## Competing interests

The authors have no financial interest to declare. There is no conflict of interest to declare.

## Authors’ contribution

EK: study design, patient selection, Sample collection and handling, sample analyzing via commercially available enzyme-linked immunosorbent assay kit, draft the manuscript. MA: study design, patient selection, Sample collection and handling, draft the manuscript and revising it. AH: study design, patient selection, Sample collection and handling, revising manuscript. MA: study design, sample analyzing via commercially available enzyme-linked immunosorbent assay kit, draft the manuscript and revising it. KK: study design, patient selection, Statistical analysis, draft the manuscript and revising it. SM: study design, patient selection, Sample collection and handling, draft the manuscript and revising it. AN: study design, patient selection, Sample collection and handling, draft the manuscript and revising it. MB: sample analyzing via commercially available enzyme-linked immunosorbent assay kit, draft the manuscript and revising it. HH: patient selection, Sample collection and handling, analysis and interpretation of data, draft the manuscript and revising it. LK: patient selection, Sample collection and handling, analysis and interpretation of data, draft the manuscript and revising it. MRJ: analysis and interpretation of data , draft the manuscript and revising it. MM: study design, patient selection, Sample collection and handling, sample analyzing via commercially available enzyme-linked immunosorbent assay kit, Statistical analysis, analysis and interpretation of data, draft the manuscript and revising it. All authors read and approved the final manuscript.

## References

[B1] Moreno RP Metnitz B Adler L Hoechtl A Bauer P Metnitz PGH Sepsis mortality prediction based on predisposition, infection and response Intensive Care Med 2008 34 496 504 10.1007/s00134-007-0943-1 18060541

[B2] Vesteinsdottir E Karason S Sigurdsson SE Gottfredsson M Sigurdsson GH Severe sepsis and septic shock: a prospective population-based study in Icelandic intensive care units Acta Anaesthesiol Scand 2011 55 722 731 10.1111/j.1399-6576.2011.02437.x 21480832

[B3] Caille V Bossi P Grimaldi D Vieillard-Baro A [Physiopathology of severe sepsis] Presse Med 2004 33 256 261 discussion 269 10.1016/S0755-4982(04)98551-X 15029017

[B4] Bozza FA Salluh JI Japiassu AM Soares M Assis EF Gomes RN Bozza MT Castro-Faria-Neto HC Bozza PT Cytokine profiles as markers of disease severity in sepsis: a multiplex analysis Critical Care 2007 11 R49 10.1186/cc5783 17448250 PMC2206478

[B5] Mousavi S Mojtahedzadeh M Abdollahi M Place of iron chelators like desferrioxamine and deferasirox in management of hyperoxia-induced lung injury: A systematic review Int J Pharmacol 2010 6 326 337

[B6] Shiehmorteza M Ahmadi A Abdollahi M Nayebpour M Mohammadi M Hamishehkar H Najafi A Pazoki M Mojtahedzadeh M Recombinant human erythropoietin reduces plasminogen activator inhibitor and ameliorates pro-inflammatory responses following trauma DARU J Pharmaceut Sci 2011 19 159 165 PMC3232093 22615653

[B7] Soltan-Sharifi MS Mojtahedzadeh M Najafi A Khajavi MR Rouini MR Moradi M Mohammadirad A Abdollahi M Improvement by N-acetylcysteine of acute respiratory distress syndrome through increasing intracellular glutathione, and extracellular thiol molecules and anti-oxidant power: evidence for underlying toxicological mechanisms Hum Exp Toxicol 2007 26 697 703 10.1177/0960327107083452 17984140

[B8] Gogos CA Drosou E Bassaris HP Skoutelis A Pro-versus anti-inflammatory cytokine profile in patients with severe sepsis: a marker for prognosis and future therapeutic options J Infect Dis 2000 181 176 180 10.1086/315214 10608764

[B9] Dimopoulou I Orfanos S Kotanidou A Livaditi O Giamarellos-Bourboulis E Athanasiou C Korovesi I Sotiropoulou C Kopterides P Ilias I Plasma pro-and anti-inflammatory cytokine levels and outcome prediction in unselected critically ill patients Cytokine 2008 41 263 267 10.1016/j.cyto.2007.11.019 18191577

[B10] Hamishehkar HB MT Abdollahi M Ahmadi A Ahmadi A Mahmoodpour Mirjalili MR Abrishami R Khoshayand MR Eslami K Kanani M Baeeri M Mojtahedzadeh M Identification of enhanced cytokine generation following sepsis. Dream of magic bullet for mortality prediction and therapeutic evaluation DARU 2010 18 155 162 22615611 PMC3304360

[B11] Keh D Goodman S Sprung CL Corticosteroid therapy in patients with severe sepsis and septic shock Semin Respir Crit Care Med 2004 25 713 719 10.1055/s-2004-860985 16088513

[B12] Minneci PC Deans KJ Natanson C Corticosteroid therapy for severe sepsis and septic shock JAMA 2009 302 1643 author reply 1644–1645 19843895 10.1001/jama.2009.1480

[B13] Panacek EA Marshall JC Albertson TE Johnson DH Johnson S MacArthur RD Miller M Barchuk WT Fischkoff S Kaul M Efficacy and safety of the monoclonal anti-tumor necrosis factor antibody F(ab')2 fragment afelimomab in patients with severe sepsis and elevated interleukin-6 levels Crit Care Med 2004 32 2173 2182 15640628 10.1097/01.ccm.0000145229.59014.6c

[B14] Reinhart K Menges T Gardlund B Harm Zwaveling J Smithes M Vincent JL Tellado JM Salgado-Remigio A Zimlichman R Withington S Randomized, placebo-controlled trial of the anti-tumor necrosis factor antibody fragment afelimomab in hyperinflammatory response during severe sepsis: The RAMSES Study Crit Care Med 2001 29 765 769 10.1097/00003246-200104000-00015 11373466

[B15] Knaus WA Harrell FE Jr LaBrecque JF Wagner DP Pribble JP Draper EA Fisher CJ Jr Soll L Use of predicted risk of mortality to evaluate the efficacy of anticytokine therapy in sepsis. The rhIL-1ra Phase III Sepsis Syndrome Study Group Crit Care Med 1996 24 46 56 10.1097/00003246-199601000-00010 8565538

[B16] Suputtamongkol Y Intaranongpai S Smith MD Angus B Chaowagul W Permpikul C Simpson JA Leelarasamee A Curtis L White NJ A double-blind placebo-controlled study of an infusion of lexipafant (Platelet-activating factor receptor antagonist) in patients with severe sepsis Antimicrob Agents Chemother 2000 44 693 696 10.1128/AAC.44.3.693-696.2000 10681340 PMC89748

[B17] Vincent JL Spapen H Bakker J Webster NR Curtis L Phase II multicenter clinical study of the platelet-activating factor receptor antagonist BB-882 in the treatment of sepsis Crit Care Med 2000 28 638 642 10.1097/00003246-200003000-00006 10752807

[B18] Angstwurm MW Engelmann L Zimmermann T Lehmann C Spes CH Abel P Strauss R Meier-Hellmann A Insel R Radke J Selenium in Intensive Care (SIC): results of a prospective randomized, placebo-controlled, multiple-center study in patients with severe systemic inflammatory response syndrome, sepsis, and septic shock Crit Care Med 2007 35 118 126 10.1097/01.CCM.0000251124.83436.0E 17095947

[B19] Valenta J Brodska H Drabek T Hendl J Kazda A High-dose selenium substitution in sepsis: a prospective randomized clinical trial Intensive Care Med 2011 37 808 815 10.1007/s00134-011-2153-0 21347869

[B20] Abrishami RA A Abdollahi M Moosivand A Khalili H Najafi A Gholami K Hamishehkar H Yazdi AP Mojtahedzadeh M Comparison the inflammatory effects of early supplemental parenteral nutrition plus enteral nutrition versus enteral nutrition alone in critically ill patientsdaru 2010 18 103 106 22615602 PMC3304373

[B21] Bernard GR Drotrecogin alfa (activated) (recombinant human activated protein C) for the treatment of severe sepsis Crit Care Med 2003 31 S85 93 10.1097/00003246-200301001-00012 12544981

[B22] Hack CE Ogilvie AC Eisele B Jansen PM Wagstaff J Thijs LG Initial studies on the administration of C1-esterase inhibitor to patients with septic shock or with a vascular leak syndrome induced by interleukin-2 therapy Prog Clin Biol Res 1994 388 335 357 7831367

[B23] Peng Z Pai P Han-Min W Jun Z Hong-Bao L Rong L Chen H Evaluation of the effects of pulse high-volume hemofiltration in patients with severe sepsis: a preliminary study Int J Artif Organs 2010 33 505 511 20872345 10.1177/039139881003300801

[B24] Patwardhan B Gautam M Botanical immunodrugs: scope and opportunities Drug Discov Today 2005 10 495 502 10.1016/S1359-6446(04)03357-4 15809195 PMC7128543

[B25] Paydary K Emamzadeh-Fard S Khorshid H Kamali K Seyed AS Mohraz M Safety and Efficacy of Setarud (IMOD (TM)) Among People Living with HIV/AIDS. A Review Recent patents on anti-infective drug discovery 2012 7 66 72 10.2174/157489112799829756 22353002

[B26] Mohammadirad AK-KH Gharibdoost F Abdollahi M etarud (IMODTM) as a multiherbal drug with promising benefits in animal and human studies: A comprehensive review of biochemical and cellular evidences Asian J Anim Vet Adv 2011 6 1185 1192 10.3923/ajava.2011.1185.1192

[B27] Mohraz M S B F M K K c R Evaluation of safety and efficacy of IMOD inb patients with HIV/AIDS Iranian J Allergy Asthma Immunol 2007 6 10

[B28] Mohraz M Khairandish P Kazerooni PA Davarpanah MA Shahhosseiny MH Mahdavian B Vaziry S Shahriary S Kamali K Khorshid KHR A clinical trial on the efficacy of IMOD in AIDS patients Daru-J Pharmaceut Sci 2009 17 277 284

[B29] Keirandish PMM khorram Khorshid H Heshmat R Gharibdust F Aphase I clinical trial to determine the maximum tolerated dose and toxicity of IMOD in HIV infected asymptomatic patiets in Iran Iranian J Allergy,Asthma Immunol 2007 6 5

[B30] Mahmoodpoor A Eslami K Mojtahedzadeh M Najafi A Ahmadi A Dehnadi-Moghadam A Mohammadirad A Baeeri M Abdollahi M Examination of Setarud (IMOD (TM)) in the management of patients with severe sepsis Daru-Journal of Faculty of Pharmacy 2010 18 23 28 PMC3232081 22615589

[B31] Hicks P Cooper DJ The Surviving Sepsis Campaign: International guidelines for management of severe sepsis and septic shock: 2008 Crit Care Resusc 2008 10 8 18304010

[B32] Dombrovskiy VY Martin AA Sunderram J Paz HL Rapid increase in hospitalization and mortality rates for severe sepsis in the United States: a trend analysis from 1993 to 2003 Crit Care Med 2007 35 1244 1250 10.1097/01.CCM.0000261890.41311.E9 17414736

[B33] Busund R Koukline V Utrobin U Nedashkovsky E Plasmapheresis in severe sepsis and septic shock: a prospective, randomised, controlled trial Intensive care medicine 2002 28 1434 1439 10.1007/s00134-002-1410-7 12373468

[B34] Dhainaut JF Laterre PF LaRosa SP Levy H Garber GE Heiselman D Kinasewitz GT Light RB Morris P Schein R The clinical evaluation committee in a large multicenter phase 3 trial of drotrecogin alfa (activated) in patients with severe sepsis (PROWESS): Role, methodology, and results Critical care medicine 2003 31 2291 2301 10.1097/01.CCM.0000085089.88077.AF 14501959

[B35] Laterre PF Levy H Clermont G Ball DE Garg R Nelson DR Dhainaut JF Angus DC Hospital mortality and resource use in subgroups of the Recombinant Human Activated Protein C Worldwide Evaluation in Severe Sepsis (PROWESS) trial* Critical care medicine 2004 32 2207 15640632 10.1097/01.ccm.0000145231.71605.d8

[B36] Wiedermann C Kaneider N A meta-analysis of controlled trials of recombinant human activated protein C therapy in patients with sepsis BMC emergency medicine 2005 5 7 10.1186/1471-227X-5-7 16225672 PMC1266358

[B37] Reshetnikov EA Baranov GA Chuvanov MV Skalozub O Reshetnikov EA Baranov GA Chuvanov MV Skalozub OI [Immunotherapy in complex treatment of surgical sepsis] Khirurgiia (Mosk) 2008 7 11 14 18833157

[B38] Li W Ashok M Li J Yang H Sama AE Wang H A major ingredient of green tea rescues mice from lethal sepsis partly by inhibiting HMGB1 PLoS One 2007 2 e1153 10.1371/journal.pone.0001153 17987129 PMC2048740

[B39] Wheeler DS Lahni PM Hake PW Denenberg AG Wong HR Snead C Catravas JD Zingarelli B The green tea polyphenol epigallocatechin-3-gallate improves systemic hemodynamics and survival in rodent models of polymicrobial sepsis Shock 2007 28 353 359 10.1097/shk.0b013e3180485823 17545942

[B40] Abedi-valugerdi MAN Khorram Khorsid H Tavasoti Kheiry M Garibdust F In vivo and ex vivo immune response on herbal drug IMOD in animal experiments Iranian J Allergy Asthma Immunol 2007 6 9

[B41] Rezvanfar MA Ahmadi A Baeeri M Abdollahi M Inhibition of tumor necrosis factor-alpha and nitrosative/oxidative stresses by Setarud (IMOD®); a molecular mechanism of protection against letrozole-induced polycystic ovary Toxicology Letters 2011 205 S246

[B42] Bauer M [The physiopathology of sepsis. Current concepts] Anaesthesist 1996 45 312 322 10.1007/s001010050266 8702048

[B43] Cavazzoni SLZ Dellinger RP Hemodynamic optimization of sepsis-induced tissue hypoperfusion Critical Care 2006 10 S2 10.1186/cc4829 PMC3226124 17164014

[B44] Levy R Mitochondrial dysfunction, bioenergenic impairment, and metabolic down-regulation in sepsisshock 2007 28 24 28 10.1097/01.shk.0000235089.30550.2d 17483747

[B45] Khairandish P Mohraz M Farzamfar B Abdollahi M Shahhosseiny MH Madani H Sadeghi B Heshmat R Gharibdoust F Khorram-Khorshid HR Preclinical and phase 1 clinical safety of Setarud (IMOD (TM)), a novel immunomodulator Daru-J Pharmaceut Sci 2009 17 148 156

